# COVID-19 musculoskeletal involvement in children

**DOI:** 10.3389/fped.2023.1200877

**Published:** 2023-05-19

**Authors:** Chiara Giraudo, Giulia Fichera, Lucia Pilati, Anna Laura Cortinovis, Celeste Cavallin, Sofia Bertin, Monica Zuliani, Diego Cecchin

**Affiliations:** ^1^ Department of Medicine—DIMED, University of Padova, Padova, Italy; ^2^ Pediatric Radiology Unit, University Hospital of Padova, Padova, Italy

**Keywords:** COVID-19, children, musculoskeletal system, myositis, arthritis

## Abstract

Since the early phases of the COVID-19 pandemic, it has become clear that children are affected by mild respiratory symptoms rather than the critical pneumonia typical in adults. Nevertheless, it took longer to understand that pediatric patients with SARS-COV2 may develop a severe multisystem inflammatory response (a.k.a. multisystem inflammatory syndrome in children (MIS-C)), which can include musculoskeletal symptoms, and/or arthritis and myositis independently from MIS-C. Diagnostic imaging significantly contributed to the assessment of pulmonary disease due to COVID-19 but it has been rarely applied to evaluate musculoskeletal involvement in children with or without previous rheumatic diseases. Despite the paucity of radiological literature, muscle edema at magnetic resonance and synovitis at ultrasound have been described. Further use of diagnostic imaging for children with articular and muscular symptoms due to COVID-19 is strongly encouraged.

## Introduction

If at the beginning of the SARS-COV2 pandemic all attention was centered on respiratory symptoms, as time passed by, given also the beneficial effects of massive vaccination efforts that led to less severe pneumonia and improved the survival rates, it became clear that although the lungs were the main target organ, the infection acted at a systemic level with direct or indirect multiorgan involvement (1–3). Indeed, the occurrence of neurological, gastrointestinal, vascular, and musculoskeletal symptoms has been widely reported in the acute phase as well as part of the long-COVID syndrome (4–7). Regarding muscles and joints, arthritis and myositis have been referred in both adults and children (8–11). For children, it was clear since the early phases of the COVID-19 spread that they often have mild respiratory symptoms but it took a while longer to discover that, on the other hand, they may undergo a severe multisystem inflammatory response (a.k.a. multisystem inflammatory syndrome in children MIS-C)) (12). This syndrome may include musculoskeletal symptoms although not listed among the main diagnostic criteria (12, 13).

Diagnostic imaging significantly contributed in understanding the chameleonic behavior of COVID-19 not only in terms of pulmonary involvement with the development of dedicated scores and advanced quantitative analyses but also for the extrapulmonary disease (14–22). Several studies focused on the occurrence of arthritis and myositis especially in adults and some reviews already summarized the evidence in this group of patients (8, 23). Nevertheless, to the best of our knowledge, a comprehensive review addressing the musculoskeletal involvement in children and taking into account the role of imaging and patients with known rheumatic diseases has not been performed yet.

Thus, our aim is to review the main musculoskeletal symptoms related to COVID-19 infection in children, highlighting the findings at imaging, the risks for children with previous diagnosis of inflammatory disease, and addressing the areas that require further investigation to fully understand the systemic involvement caused by SARS2-COV infections and better target the therapeutic approach.

## Myositis

Benign acute myositis in children is a rare, transient inflammatory process usually occurring after a viral infection with a median age of 6–9 years and male predominance (24). Given the self-limiting behavior of this condition, radiological imaging is not part of the usual diagnostic workflow, although, for instance, Kawarai et al. demonstrated by magnetic resonance (MR) muscle edema and the full recovery after 30 days in a 15-year-old girl after influenza A infection (25).

**Figure 1:**
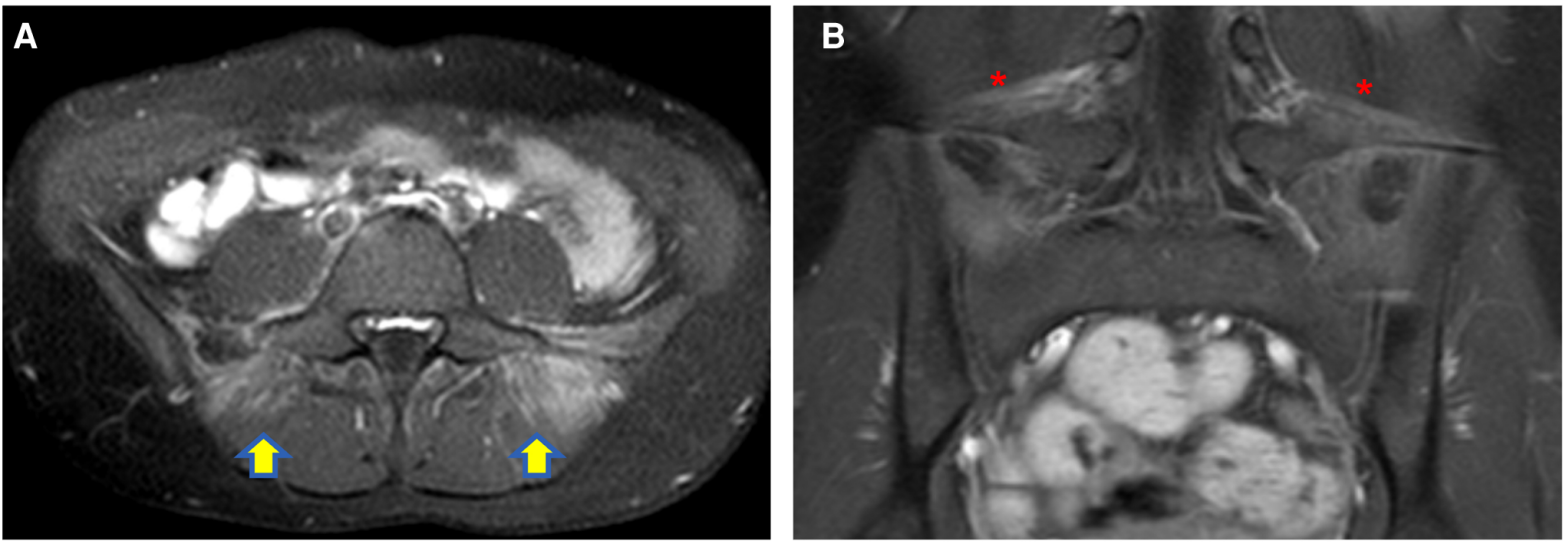
Axial (**A**) and coronal post-contrast fat-sat T1W (**B**) images of a 12-year-old girl with COVID-19 infection complaining of back pain, well demonstrating myositis of the paravertebral muscle [yellow arrows in (**A**)] and bilateral enthesitis of the iliolumbar ligaments [red asterisks in (**B**)].

Similarly, myositis, which can be easily detected as muscle edema at MR has been reported in children with COVID-19 (26–28) (Figure 1).

The overall mechanism behind this expression of the disease is still unknown, but it might be related either to a direct effect of the virus or associated with an autoimmune response (28, 29). Movahedi and Ziaee, reporting an increase of juvenile dermatomyositis in their center during the current pandemic, hypothesize three possible mechanisms: (i) a type 1 IFN pathway dysregulation because of the Myxovirus resistance protein A, a type I interferon-inducible protein expressed in response to viral infection including SARS-CoV-2; (ii) post-viral myositis with diffuse or multifocal muscle pain and/or rhabdomyolysis; (iii) dermatomyositis-like syndrome, mimicking a dermatomyositis (30).

Furthermore, Qian and Xu in their review described the common pathogenetic mechanism between COVID-19 and dermatomyositis (31). In fact, COVID-19 and dermatomyositis share three immunogenic linear epitopes with high sequence consistency. The innate and adaptive immune response triggered by the entrance of SARS-COV-2 in human cells via the angiotensin-converting enzyme 2 causes the production of cytokines, which then induce pulmonary and muscle injuries (31).

Independent from the mechanism, although most of the children with myositis referred symptoms of the extremities, such as the case described by Liquidano-Perez et al. with myositis of the thighs and gluteal muscle at MR, a few cases of orbital myositis due to COVID-19 have also been described (20, 26, 28). For example, Miglani et al. reported the occurrence of orbital myositis in a 12-year-old boy with bilateral enlargement of multiple orbital muscles using CT (32). The patient was then successfully treated with steroids (i.e., intravenous methylprednisolone 1 mg/kg followed by oral prednisone 60 mg daily for 1 week then tapered over 6 weeks) and fully recovered in 1 week. Eleiwa et al. described the case of a 10-year-old boy with left-sided proptosis with enlargement of the left lateral rectus muscle at MR (27). Steroid treatment was beneficial also in this patient with complete resolution at the MR of follow-up after 14 days (27).

Overall, it should not be overlooked that fatal cases of rhabdomyolysis have also been reported, such as the one of a 16-year-old boy admitted to the hospital for fever, sore throat, and myalgia who died a few days later (11, 33).

### Myositis in MIS-C

Although, as mentioned above, the MIS-C clinical criteria do not include musculoskeletal symptoms, arthralgia, arthritis, myositis, and bone marrow infiltration have been reported in children with this multisystem syndrome (34, 35).

Several cases of myositis in children with MIS-C have been reported in the literature, but diagnostic imaging has been rarely applied (9, 10). For instance, in a 12-year-old African girl, SARS-CoV-2-positive and satisfying the criteria of MIS-C, admitted to the tertiary academic hospital of Johannesburg with severe acute inflammatory myositis complicated by rhabdomyolysis and then successfully treated with immunoglobulin and steroid, no radiological examinations of the muscles have been performed (9).

Similarly, Fabi et al. described severe rhabdomyolysis that affected a 6-year-old African girl suggesting that the muscle injury resembles the one characterizing necrotizing autoimmune myositis, but also in this case, no imaging was reported (10).

While efforts have been devoted in trying to distinguish between Kawasaki disease and MIS-C due to COVID-19, still much has to be understood to fully understand whether muscular symptoms are part of the multisystemic inflammatory syndrome or just a localized expression of the disease (36). Probably, the occurrence of muscular symptoms in children satisfying the MIS-C criteria should be seen as part of the multisystem involvement of this inflammatory entity, and in case the criteria are not fulfilled, myositis should be addressed *per se* although further evidence should be acquired to better address the potential overlap of these disease expressions.

## Arthritis and arthralgia

**Figure 2:**
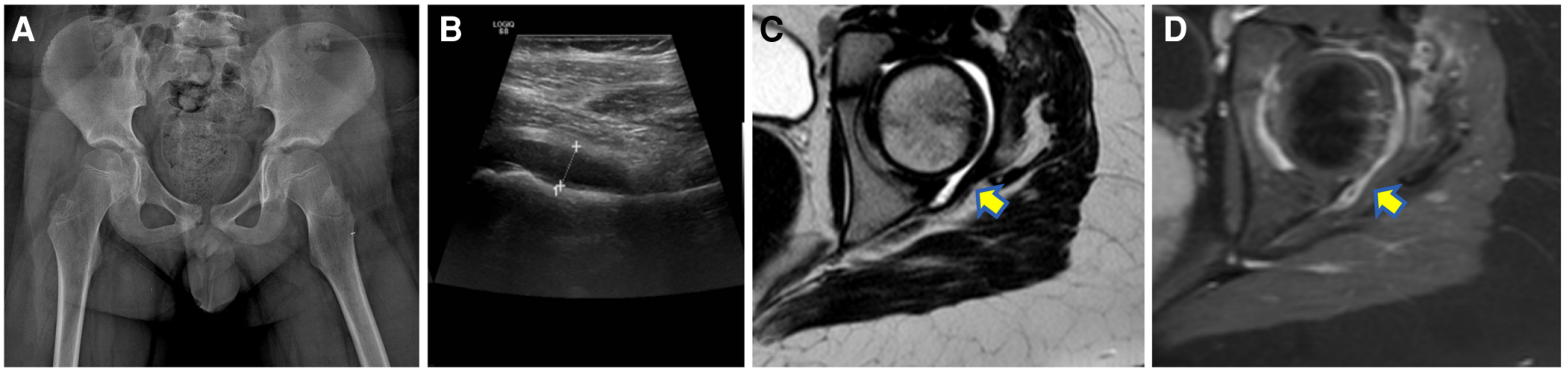
A 10-year-old boy with left hip pain 2 weeks after a mild respiratory COVID-19 infection with negative radiographs (**A**) but joint effusion at ultrasound (**B**) then confirmed at the MR [yellow arrow on the axial T2W turbo spin echo sequence in (**C**)] which also demonstrated synovial enhancement after contrast medium injection [yellow arrow in (**D**)].

Reactive arthritis usually occurs 4–6 weeks after an infection, and it is well known that in children it may have a viral etiology (e.g., Rubella, Parvovirus B19, and Hepatitis B) in around 1% of the cases (37). Several episodes of reactive arthritis due to COVID-19 have been described in the pediatric population (38–40). For instance, Sinaei et al. published two cases (8-year-old boy and 6-year-old girl) characterized by involvement of the hip joint, investigated by MRI and US, respectively, both showing joint effusion. These two children had mild or not significant respiratory symptoms and fever and were successfully treated for the arthritis with nonsteroidal anti-inflammatory drugs recovering in a short interval of time without sequelae (39). Postinfectious arthritis is usually acute and responsive to treatment if not associated with MIS-C. Joint effusion and tenosynovitis have mainly been reported at imaging in children with COVID-19 (40, 41) (Figure 2). Houshmand et al. described the clinical and radiological signs of arthritis in both knees and in the right elbow of a 10-year-old boy with COVID-19 (40). Dutta et al. reported the case of a 14-year-old male with polyarthritis (right elbow, bilateral knees, and ankles) due to a previous COVID-19 infection (i.e., 3 weeks before). In this child, the ultrasound demonstrated knee joint effusion without tenosynovitis, which fully resolved after steroids (41). Finally, a case of dactylitis related to COVID-19 affecting the second, fourth, and fifth metatarsophalangeal joints in a 16-year-old girl has been published (42). The inflammatory process was successfully treated with naproxen for 5 days. It is unfortunate that in this case, diagnostic imaging has not been reported, as it may have contributed to a better understanding of the superficial and deep involvement.

### Arthritis and arthralgia in MIS-C

As for myositis, joint involvement, such as arthralgia and/or arthritis, has been frequently described in association with MIS-C. For example, Crivelenti et al. published the case of a 11-year-old girl with MIS-C who developed severe myalgia and arthritis in the ankles, knees, elbows, wrists, and interphalangeal joints 3 days after the onset of fever (43). After hospital discharge, during the follow-up, joint pain persisted with evidence of synovitis at ultrasound. Given the persistence of articular symptoms for 6 weeks, she was treated with steroids with clinical improvement. Five months after the infection, synovial thickening at ultrasound, morning stiffness, and high erythrocyte sedimentation rate levels were still evident suggesting chronic arthritis (43). Moreover, the overlap of MIS-C and systemic-onset juvenile idiopathic arthritis has been described in a 12-month-old child admitted to the emergency room for high fever and multiple joint swelling partially responsive to antipyretics (44).

## Osteonecrosis and overall bone marrow involvement

In adults, given also the inclusion of steroids for COVID-19 infection, the occurrence of bone marrow edema and avascular necrosis have been reported, but so far, no pediatric cases have been published (8, 45). In children, a few histological studies on children with MIS-C demonstrated that the erythroid and megakaryocytic lineages were affected and there was hemophagocytosis (46, 47). The alteration of the megakaryocytopoiesis seems to be a peculiar feature associated with COVID-19, which also allows the distinction from secondary hemophagocytic lymphohistiocytosis (46). Finally, regarding bone marrow infiltration, there was a fatal case of COVID-19 that induced bone marrow aplasia probably as part of the cytokine cascade in a 6-year-old girl who was otherwise healthy before the infection (48). Unfortunately, to the best of our knowledge, there are no studies that focused on the histopathological–radiological correlation of bone marrow findings in children. Skeletal MR could provide additional insights for this type of infiltration. Nevertheless, we call for studies addressing this topic to better understand the behavior of the disease in this compartment and improve the overall diagnostic and therapeutic management of children with COVID-19 with a systemic approach.

## Chondritis

Collins et al. described the case of an immunocompetent 11-year-old boy with a long-lasting, severe costochondritis associated with COVID-19 infection unresponsive to the conventional treatment of non-steroidal anti-inflammatory drugs (NSAIDs) or steroids but then successfully treated with colchicine (49). In adults, Tiez's syndrome in an immunocompetent patient as well as costochondritis with chest wall abscess due to secondary infections in patients with COVID-19 treated with immunotherapy (e.g., tocilizumab) have also been reported and characterized at imaging with evidence at MR of edema of the affected joint in the former and a fluid collection with peripheral enhancement in the latter (50–52).

## COVID-19 in children with previously diagnosed rheumatic diseases or preexisting neuromuscular diseases

Two national studies (i.e., in Germany and France, respectively) demonstrated that children and adolescents with rheumatic diseases and COVID-19 have an excellent prognosis (53, 54). Clemente et al. in a multicenter study in Spain concluded that comorbidities (e.g., diabetes) and the use of glucocorticoids were associated with a higher risk of hospitalization in children with rheumatic diseases and COVID-19 (55). Partially in line with this evidence, a study based on the European Alliance of Associations for Rheumatology COVID-19 Registry, the Childhood Arthritis and Rheumatology Research Alliance (CARRA) Registry, and the CARRA-sponsored COVID-19 Global Paediatric Rheumatology Database demonstrated that children and young people with severe systemic rheumatic diseases and obesity were more likely to be hospitalized (56). A recent meta-analysis investigating the risk of COVID-19 infection and the outcome in adults and children with rheumatic diseases demonstrated that patients with rheumatic and musculoskeletal diseases (RMDs) have higher rates of SARS-CoV-2 infection and an increased mortality rate, but it has to be highlighted that only 1% and 14% of the studies focused on children only or included pediatric patients; thus, this evidence may just partially apply to this group of patients (57). Regarding juvenile idiopathic arthritis, Hügle et al. demonstrated in five children in remission or with inactive disease on medication that flares can occur after infection with SARS-CoV-2 (58). Quintana-Ortega et al. reported the fatal case of a 11-year-old girl with anti-melanoma differentiation-associated gene 5 juvenile dermatomyositis (anti-MDA5 JDM) with rapidly progressive interstitial lung disease, further complicated by SARS-CoV-2 infection (59). Unfortunately, the above-mentioned studies did not include any finding at musculoskeletal imaging to evaluate the clinical performance of this group of patients.

A few studies addressed COVID-19 in children with preexisting neuromuscular diseases (60–62). Attention, especially in the early phase of the pandemic, has been mainly devoted to the risk of severe COVID-19 complications in case of cardiorespiratory involvement for instance in patients with myasthenic syndromes (60, 63). Exacerbations of myasthenia gravis have been reported in adults with COVID-19 (60). A study including children with type 1 and 2 spinal muscular atrophy showed that the protective factor related to the young age of this group of patients outbalances the risk factor related to the preexisting neuromuscular disease (62). Regarding muscular dystrophies, it seems that they do predispose to more severe SARS-COV-2 infections, and the main impact of the pandemic on this group of patients seems to be associated with a reduced access to care including, in particular, the limited admission to rehabilitation centers where to perform regular physical exercises (60). Similar evidence emerged for children with peripheral nerve disorders such as Charcot–Marie–Tooth (64).

## Further Research

The above-mentioned literature studies show that the musculoskeletal system can be affected by the infection of COVID-19 also in children. Although most of the symptoms are self-limiting or respond to treatment, we call for further application of diagnostic imaging in this group of patients. Indeed, the application of MR or ultrasound, even bedside in case of hospitalized children, may provide a deeper insight into the features of this disease expression characterizing joint and muscle involvement (65). Follow-up after treatment is also recommended to better understand the timing of remission and complete resolution aiming to optimize treatment and potential rehabilitation programs.

## Conclusions

In conclusion, in children, COVID-19 infection may induce musculoskeletal symptoms beyond MIS-C, which can be detected at imaging. So far, diagnostic imaging has been rarely applied to investigate these findings, but we strongly encourage its use especially in terms of ultrasound and MR to better understand the extension and behavior of this type of disease expression at diagnosis and during follow-up aiming to improve the overall therapeutic management.

## References

[1] Guan WJ Ni ZY Hu Y Liang WH Ou CQ He JX Clinical characteristics of coronavirus disease 2019 in China. N Engl J Med. (2020) 382:1708–20. 10.1056/NEJMoa2002032 32109013 PMC7092819

[2] Gandhi RT Lynch JB Carlos del Rio MPH. Mild or moderate COVID-19. N Engl J Med. (2020) 383:1757–66. 10.1056/NEJMcp2009249 32329974

[3] Calabrese F Pezzuto F Fortarezza F Boscolo A Lunardi F Giraudo C Machine learning-based analysis of alveolar and vascular injury in SARS-CoV-2 acute respiratory failure. J Pathol. (2021) 254:173–84. 10.1002/path.5653 33626204 PMC8014445

[4] Kang D Park SH Oh C Kim YJ Kim JB Park SH Prevalence and prognosis of acute pancreatitis in critically ill patients with COVID-19. Hepatobiliary Pancreat Dis Int. (2023). S1499-3872(23)00038-3. 10.1016/j.hbpd.2023.03.004. Online ahead of print PMC10017175 36973110

[5] Ancona G Alagna L Alteri C Palomba E Tonizzo A Pastena A Gut and airway microbiota dysbiosis and their role in COVID-19 and long-COVID. Front Immunol. (2023) 14:1080043. 10.3389/fimmu.2023.1080043 36969243 PMC10030519

[6] Ellul MA Benjamin L Singh B Lant S Michael BD Easton A Neurological associations of COVID-19. Lancet Neurol. (2020) 9:767–83. 10.1016/S1474-4422(20)30221-0 PMC7332267 32622375

[7] Xu E Xie Y Al-Aly Z. Long-term neurologic outcomes of COVID-19. Nat Med. (2022) 28:2406–15. 10.1038/s41591-022-02001-z 36138154 PMC9671811

[8] Omar IM Weaver JS Samet JD Serhal AM Mar WA Taljanovic MS. Musculoskeletal manifestations of COVID-19: currently described clinical symptoms and multimodality imaging findings. Radiographics. (2022) 42:1415–32. 10.1148/rg.220036 35867593 PMC9341171

[9] Cassim F Soni AJ Murphy S. Severe acute inflammatory myositis and rhabdomyolysis in paediatric SARS-CoV-2-associated MIS-C (multisystem inflammatory syndrome in children). BMJ Case Rep. (2021) 14:e243112. 10.1136/bcr-2021-243112 34373240 PMC8354289

[10] Fabi M Guida F Pierantoni L Greco L di Luca D Lanari M. Severe rhabdomyolysis in a child with multisystem inflammatory syndrome: an autoimmune mechanism? Pediatr Neurol. (2021) 124:11–2. 10.1016/j.pediatrneurol.2021.07.015 34507248 PMC8349362

[11] Anwar H Al Lawati A. Adolescent COVID-19-associated fatal rhabdomyolysis. J Prim Care Community Health. (2020) 11:2150132720985641. 10.1177/2150132720985641 33357150 PMC7780181

[12] Feldstein LR Rose EB Horwitz SM Collins JP Newhams MM Son MBF Multisystem inflammatory syndrome in U.S. children and adolescents. N Engl J Med. (2020) 383:334–46. 10.1056/NEJMoa2021680 32598831 PMC7346765

[13] Cattalini M Taddio A Bracaglia C Cimaz R Paolera SD Filocamo G Childhood multisystem inflammatory syndrome associated with COVID-19 (MIS-C): a diagnostic and treatment guidance from the rheumatology study group of the Italian society of pediatrics. Ital J Pediatr. (2021) 47:24. 10.1186/s13052-021-00980-2 33557873 PMC7868856

[14] Helms J Kremer S Merdji H Clere-Jehl R Schenck M Kummerlen C Neurologic features in severe SARS-CoV-2 infection. N Engl J Med. (2020) 382:2268–70. 10.1056/NEJMc2008597 32294339 PMC7179967

[15] Bhayana R Som A Li MD Carey DE Anderson MA Blake MA Abdominal imaging findings in COVID-19: preliminary observations. Radiology. (2020) 297:E207–15. 10.1148/radiol.2020201908 32391742 PMC7508000

[16] Kremer S Lersy F de Sèze J Ferré JC Maamar A Carsin-Nicol B Brain MRI findings in severe COVID-19: a retrospective observational study. Radiology. (2020) 297:E242–51. 10.1148/radiol.2020202222 32544034 PMC7301613

[17] Giraudo C Fichera G Motta R Guarnieri G Plebani M Pelloso M It’s not just the lungs: COVID-19 and the misty mesentery sign. Quant Imaging Med Surg. (2021) 11:2201–3. 10.21037/qims-20-1406 33937000 PMC8047374

[18] Fichera G Stramare R De Conti G Motta R Giraudo C. It’s not over until it’s over: the chameleonic behavior of COVID-19 over a six-day period. Radiol Med. (2020) 125:514–16. 10.1007/s11547-020-01203-0 32350796 PMC7189828

[19] Cocconcelli E Castelli G Onelia F Lavezzo E Giraudo C Bernardinello N Disease severity and prognosis of SARS-CoV-2 infection in hospitalized patients is not associated with viral load in nasopharyngeal swab. Front Med. (2021) 8:714221. 10.3389/fmed.2021.714221 PMC8460755 34568371

[20] Giraudo C Cavaliere A Fichera G Weber M Motta R Pelloso M Validation of a composed COVID-19 chest radiography score: the CARE project. ERJ Open Res. (2020) 6:359–2020. 10.1183/23120541.00359-2020 PMC7682711 33263058

[21] Russo E Tagliafico AS Derchi L Bignotti B Tosto S Martinoli C Role of renal parenchyma attenuation and perirenal fat stranding in chest CT of hospitalized patients with COVID-19. J Clin Med. (2023) 12:929. 10.3390/jcm12030929 36769577 PMC9918001

[22] Giraudo C Librizzi G Fichera G Motta R Balestro E Calabrese F Reduced muscle mass as predictor of intensive care unit hospitalization in COVID-19 patients. PLoS One. (2021) 16:e0253433. 10.1371/journal.pone.0253433 34138945 PMC8211180

[23] Kanmaniraja D Kurian J Holder J Gunther MS Chernyak V Hsu K Review of COVID-19, part 1: abdominal manifestations in adults and multisystem inflammatory syndrome in children. Clin Imaging. (2021) 80:88–110. 10.1016/j.clinimag.2021.06.025 34298343 PMC8223038

[24] Costa Azevedo A E Silva AC Juliana Silva C Poço Miranda S Costa M Martinho I. Benign acute childhood myositis: a 5-year retrospective study. Arch Pediatr. (2022) 29:490–93. 10.1016/j.arcped.2022.08.009 36109287

[25] Kawarai T Nishimura H Taniguchi K Saji N Shimizu H Tadano M Magnetic resonance imaging of biceps femoris muscles in benign acute childhood myositis. Arch Neurol. (2007) 64:1200–01. 10.1001/archneur.64.8.1200 17698714

[26] Liquidano-Perez E García-Romero MT Yamazaki-Nakashimada M Maza-Morales M Rivas-Calderón MK Bayardo-Gutierrez B Juvenile dermatomyositis triggered by SARS-CoV-2. Pediatr Neurol. (2021) 121:26–7. 10.1016/j.pediatrneurol.2021.05.011 34126319 PMC8136292

[27] Eleiwa T Abdelrahman SN ElSheikh RH Elhusseiny AM. Orbital inflammatory disease associated with COVID-19 infection. J AAPOS. (2021) 25:232–4. 10.1016/j.jaapos.2021.04.002 33965589 PMC8099788

[28] Tekin E Akoğlu HA. From influenza to SARS-CoV-2: etiological evaluation of acute benign childhood myositis. Acta Neurol Belg. (2022) 122:1043–47. 10.1007/s13760-021-01785-0 34427875 PMC8383251

[29] Saud A Naveen R Aggarwal R Gupta L. COVID-19 and myositis: what we know so far. Curr Rheumatol Rep. (2021) 23:63. 10.1007/s11926-021-01023-9 34216297 PMC8254439

[30] Movahedi N Ziaee V. COVID-19 and myositis; true dermatomyositis or prolonged post viral myositis? Pediatr Rheumatol Online J. (2021) 19:86. 10.1186/s12969-021-00570-w 34112199 PMC8190732

[31] Qian J Xu H. COVID-19 disease and dermatomyositis: a mini-review. Front Immunol. (2022) 12:747116. 10.3389/fimmu.2021.747116 35095837 PMC8792859

[32] Miglani T Mohammed T Jensen A Bregman J. A unique case of orbital inflammatory syndrome following COVID-19 infection. J AAPOS. (2022) 26:326–8. 10.1016/j.jaapos.2022.08.267 36195133 PMC9527193

[33] Gefen AM Palumbo N Nathan SK Singer PS Castellanos-Reyes LJ Sethna CB. Pediatric COVID-19-associated rhabdomyolysis: a case report. Pediatr Nephrol. (2020) 35:1517–20. 10.1007/s00467-020-04617-0 32447505 PMC7244938

[34] Centers for Disease control and prevention. Information for Healthcare Providers about Multisystem Inflammatory Syndrome in Children (MIS-C). Available at: https://www.cdc.gov/mis/mis-c/hcp_cstecdc/index.html (Accessed March 16, 2023).

[35] Abrams JY Godfred-Cato SE Oster ME Chow EJ Koumans EH Bryant B Multisystem inflammatory syndrome in children associated with severe acute respiratory syndrome coronavirus 2: a systematic review. J Pediatr. (2020) 226:45–54.e1. 10.1016/j.jpeds.2020.08.003 32768466 PMC7403869

[36] Kostik MM Bregel LV Avrusin IS Dondurei EA Matyunova AE Efremova OS Distinguishing between multisystem inflammatory syndrome, associated with COVID-19 in children and the Kawasaki disease: development of preliminary criteria based on the data of the retrospective multicenter cohort study. Front Pediatr. (2021) 9:787353. 10.3389/fped.2021.787353 34858909 PMC8631532

[37] Plesca DA Luminos M Spatariu L Stefanescu M Cinteza E Balgradean M. Postinfectious arthritis in pediatric practice. Maedica. (2013) 8:164–9. 24371480 PMC3865125

[38] Farisogullari B Pinto AS Machado PM. COVID-19-associated arthritis: an emerging new entity? RMD Open. (2022) 8:e002026. 10.1136/rmdopen-2021-002026 36100294 PMC9471208

[39] Sinaei R Pezeshki S Parvaresh S Sinaei R Shiari R Hassas Yeganeh M Post SARS-CoV-2 infection reactive arthritis: a brief report of two pediatric cases. Pediatr Rheumatol Online J. (2021) 19:89. 10.1186/s12969-021-00555-9 34118941 PMC8196291

[40] Houshmand H Abounoori M Ghaemi R Bayat S Houshmand G. Ten-year-old boy with atypical COVID-19 symptom presentation: a case report. Clin Case Rep. (2020) 9:304–8. 10.1002/ccr3.3521 33362924 PMC7753279

[41] Dutta S Dey S Poddar A Pal P. Post-COVID reactive arthritis. Indian J Pediatr. (2022) 89:103. 10.1007/s12098-021-03992-2 34687437

[42] Salvatierra J Martínez-Peñalver D Salvatierra-Velasco L. COVID-19 related dactyitis. Joint Bone Spine. (2020) 87:660. 10.1016/j.jbspin.2020.06.009 32622040 PMC7328572

[43] Crivelenti LRMP Frazão MMN Maia MPM Gomes FHR de Carvalho LM. Chronic arthritis related to SARS-CoV-2 infection in a pediatric patient: a case report. Braz J Infect Dis. (2021) 25:101585. 10.1016/j.bjid.2021.101585 34043944 PMC8120484

[44] Waheed N Haider N Krishin J. A case of multisystem inflammatory syndrome in children presenting as systemic onset juvenile idiopathic arthritis. J Pak Med Assoc. (2022) 72:161–3. 10.47391/JPMA.11-1984 35099459

[45] Agarwala SR Vijayvargiya M Pandey P. Avascular necrosis as a part of “long COVID-19”. BMJ Case Rep. (2021) 14:e242101. 10.1136/bcr-2021-242101 34215639 PMC8256728

[46] De Ioris MA Scarselli A Bracaglia C Perrotta D Bernardi S Santilli V Common bone marrow signature in COVID-19-associated multisystem inflammatory syndrome in children: a first-wave small case series experience. Pediatr Blood Cancer. (2022) 69:e29919. 10.1002/pbc.29919 35986692 PMC9537984

[47] Octavius GS Wijaya JH Tan AO Muljono MP Chandra S Juliansen A. Autopsy findings of pediatric COVID-19: a systematic review. Egypt J Forensic Sci. (2022) 12:32. 10.1186/s41935-022-00288-0 35855892 PMC9281196

[48] Amanati A Hedayati SB Ziyaeyan M Honar A Dashtianeh R Rabiei N A fatal SARS-coronavirus-2 induced bone marrow aplasia complicated with invasive fungal infection and severe neutropenic enterocolitis. BMC Infect Dis. (2022) 22:682. 10.1186/s12879-022-07599-6 35945491 PMC9361242

[49] Collins RA Ray N Ratheal K Colon A. Severe post-COVID-19 costochondritis in children. Bayl Univ Med Cent Proc. (2021) 35:56–7. 10.1080/08998280.2021.1973274 PMC8477585 34966216

[50] Tan C Lim R Yeow M Balakrishnan T. Tietze’s syndrome post-COVID-19 infection in an adult patient. Cureus. (2022) 14:e27499. 10.7759/cureus.27499 PMC10564091 37817896

[51] Ergenç İ Şanal Toprak C Odabaşı Z. *Staphylococcus aureus costochondritis* and chest wall abscess in a COVID-19 patient treated with tocilizumab. Turk J Phys Med Rehabil. (2021) 67:382–5. 10.5606/tftrd.2021.8208 34870129 PMC8607000

[52] Gorospe-Sarasúa L Gallego-Rivera JI Muñoz-Molina GM Mirambeaux-Villalona RM Ajuria-Illarramendi O González-García A Costocondritis y espondilitis diferidas por Candida en paciente post-COVID-19 tratado previamente con corticoides, antibióticos y tocilizumab [Delayed *Candida costochondritis* and spondylitis in a post-COVID-19 patient previously treated with corticosteroids, antibiotics, and tocilizumab]. Arch Bronconeumol. (2021) 57:48–50. 10.1016/j.arbres.2020.12.002 34629649 PMC7832559

[53] Bourguiba R Kyheng M Koné-Paut I Rouzaud D Avouac J Devaux M COVID-19 infection among patients with autoinflammatory diseases: a study on 117 French patients compared with 1545 from the French RMD COVID-19 cohort: COVIMAI—the French cohort study of SARS-CoV-2 infection in patient with systemic autoinflammatory diseases. RMD Open. (2022) 8:e002063. 10.1136/rmdopen-2021-002063 35537796 PMC9091487

[54] Sengler C Eulert S Minden K Niewerth M Horneff G Kuemmerle-Deschner J Clinical manifestations and outcome of SARS-CoV-2 infections in children and adolescents with rheumatic musculoskeletal diseases: data from the national paediatric rheumatology database in Germany. RMD Open. (2021) 7:e001687. 10.1136/rmdopen-2021-001687 34312307 PMC8316693

[55] Clemente D Udaondo C de Inocencio J Nieto JC Del Río PG Fernández AG Clinical characteristics and COVID-19 outcomes in a regional cohort of pediatric patients with rheumatic diseases. Pediatr Rheumatol Online J. (2021) 19:162. 10.1186/s12969-021-00648-5 34838054 PMC8626725

[56] Kearsley-Fleet L Chang ML Lawson-Tovey S Costello R Fingerhutová Š Švestková N Outcomes of SARS-CoV-2 infection among children and young people with pre-existing rheumatic and musculoskeletal diseases. Ann Rheum Dis. (2022) 81:998–1005. 10.1136/annrheumdis-2022-222241 35338032 PMC8977459

[57] Conway R Grimshaw AA Konig MF Putman M Duarte-García A Tseng LY COVID-19 global rheumatology alliance. SARS-CoV-2 infection and COVID-19 outcomes in rheumatic diseases: a systematic literature review and meta-analysis. Arthritis Rheumatol. (2022) 74:766–75. 10.1002/art.42030 34807517 PMC9011807

[58] Hügle B Krumrey-Langkammerer M Haas JP. Infection with SARS-CoV-2 causes flares in patients with juvenile idiopathic arthritis in remission or inactive disease on medication. Pediatr Rheumatol Online J. (2021) 19:163. 10.1186/s12969-021-00653-8 34844609 PMC8628278

[59] Quintana-Ortega C Remesal A Ruiz de Valbuena M de la Serna O Laplaza-González M Álvarez-Rojas E Fatal outcome of anti-MDA5 juvenile dermatomyositis in a paediatric COVID-19 patient: a case report. Mod Rheumatol Case Rep. (2021) 5:101–7. 10.1080/24725625.2020.1832755 33019894 PMC8344517

[60] Goetschius DJ Kim Y Kumar A Paul D Naik S. A comprehensive review of neuromuscular manifestations of COVID-19 and management of pre-existing neuromuscular disorders in children. J Clin Med. (2022) 11:934. 10.3390/jcm11040934 35207206 PMC8876161

[61] Levine H Prais D Aharoni S Nevo Y Katz J Rahmani E COVID-19 in advanced Duchenne/Becker muscular dystrophy patients. Neuromuscul Disord. (2021) 31:607–11. 10.1016/j.nmd.2021.03.011 34053847 PMC8021445

[62] Natera-de Benito D Aguilera-Albesa S Costa-Comellas L Garcia-Romero M Miranda-Herrero CC Olives JR COVID-19 in children with neuromuscular disorders. J Neurol. (2021) 268:3081–85. 10.1007/s00415-020-10339-y 33387010 PMC7775833

[63] Tseng YH Chen TH. Care for patients with neuromuscular disorders in the COVID-19 pandemic era. Front Neurol. (2021) 12:607790. 10.3389/fneur.2021.607790 33841296 PMC8024582

[64] Prada V Bellone E Schenone A Grandis M. The suspected SARS-CoV-2 infection in a Charcot-Marie-Tooth patient undergoing postsurgical rehabilitation: the value of telerehabilitation for evaluation and continuing treatment. Int J Rehabil Res. (2020) 43:285–6. 10.1097/MRR.0000000000000418 32317558 PMC7273849

[65] Weber MA. Ultrasound in the inflammatory myopathies. Ann N Y Acad Sci. (2009) 1154:159–70. 10.1111/j.1749-6632.2009.04390.x 19250237

